# Comparative analysis of copy number variations in ulcerative colitis associated and sporadic colorectal neoplasia

**DOI:** 10.1186/s12885-016-2303-4

**Published:** 2016-04-14

**Authors:** B. M. Shivakumar, Sanjiban Chakrabarty, Harish Rotti, Venu Seenappa, Lakshmi Rao, Vasudevan Geetha, B. V. Tantry, Hema Kini, Rajesh Dharamsi, C. Ganesh Pai, Kapaettu Satyamoorthy

**Affiliations:** Department of Gastroenterology and Hepatology, Kasturba Medical College, Manipal University, Manipal, India; School of Life Sciences, Manipal University, Manipal, Karnataka 576104 India; Department of Pathology, Kasturba Medical College, Manipal University, Manipal, India; Department of Gastroenterology and Hepatology, Kasturba Medical College, Manipal University, Mangalore, India; Department of Pathology, Kasturba Medical College, Manipal University, Mangalore, India; Dharamsi Hospital, Chandni Chowk, Sangli, Maharashtra India

**Keywords:** Colorectal cancer, Ulcerative colitis, aCGH, Copy number variations, Quantitative RT-PCR, IHC

## Abstract

**Background:**

The incidence of and mortality from colorectal cancers (CRC) can be reduced by early detection. Currently there is a lack of established markers to detect early neoplastic changes. We aimed to identify the copy number variations (CNVs) and the associated genes which could be potential markers for the detection of neoplasia in both ulcerative colitis-associated neoplasia (UC-CRN) and sporadic colorectal neoplasia (S-CRN).

**Methods:**

We employed array comparative genome hybridization (aCGH) to identify CNVs in tissue samples of UC nonprogressor, progressor and sporadic CRC. Select genes within these CNV regions as a panel of markers were validated using quantitative real time PCR (qRT-PCR) method along with the microsatellite instability (MSI) in an independent cohort of samples. Immunohistochemistry (IHC) analysis was also performed.

**Results:**

Integrated analysis showed 10 overlapping CNV regions between UC-Progressor and S-CRN, with the 8q and 12p regions showing greater overlap. The qRT-PCR based panel of *MYC, MYCN, CCND1, CCND2, EGFR* and *FNDC3A* was successful in detecting neoplasia with an overall accuracy of 54 % in S-CRN compared to that of 29 % in UC neoplastic samples. IHC study showed that *p53* and *CCND1* were significantly overexpressed with an increasing frequency from pre-neoplastic to neoplastic stages. *EGFR* and *AMACR* were expressed only in the neoplastic conditions.

**Conclusion:**

CNVs that are common and unique to both UC-associated and sporadic colorectal neoplasm could be the key players driving carcinogenesis. Comparative analysis of CNVs provides testable driver aberrations but needs further evaluation in larger cohorts of samples. These markers may help in developing more effective neoplasia-detection strategies during screening and surveillance programs.

**Electronic supplementary material:**

The online version of this article (doi:10.1186/s12885-016-2303-4) contains supplementary material, which is available to authorized users.

## Background

Colorectal cancer (CRC) is the third most common form of cancer and the second leading cause of death among the cancers worldwide. Studies have shown that countries with medium and high human development index (HDI) are likely to show a rise in the incidence of CRC by 2030 [1–3]. While most sporadic CRC arise through the adenoma-carcinoma sequence, UC-CRC arises through inflammation-associated dysplasia-carcinoma sequence. In either situation, the cancer develops from acquiring hallmark genetic changes in the epithelium of the colon. The genetic alterations that might lead to the development of CRC in either pathway have, by tradition, been largely categorized into chromosomal instability (CIN) and microsatellite instability (MSI) [4–6].

Copy number variations (CNVs) in the cancer cell genome is one of the common mechanisms under CIN by which the expression of genes that contribute to cancer development is regulated and studying this can help in identifying tumor suppressor genes and oncogenes. CNVs are found frequently in the healthy population (common CNVs) too, but some of the CNVs associated with malignancy are known to harbor bona fide cancer-related genes [7–12]. Although genomically altered regions are very common in human cancer, it is often difficult to identify the true cancer gene in such amplicons because of the multiplicity of genes affected [13–15]. Genome-wide studies in different types of cancer, including CRC, have highlighted several important regions and genes involved in human cancer development, which have been significantly altered, by amplification or overexpression [16–19]. Therefore, the comparative identification of such altered regions and the genes within those regions and their role in cancer is essential for better understanding of the pathogenesis of cancer and also for clinical translation.

The incidence and deaths from CRC can be reduced by the early detection and removal of treatable neoplasia but for the lack of established markers specific for both established cancer and precancerous lesions [20]. Molecular stratification, combined with other strategies, may be suitable to distinguish those with preneoplastic changes from those with early neoplastic changes). Our previous study has shown that CNVs are progressively associated with the development and progression of UC to CRC [21]. With this background, we analyzed the CNVs involved in UC-progressors and S-CRC as compared to those with nonprogressors, and validated their role in a subset of samples by qRT-PCR and IHC techniques for identification of neoplasia in two of the CRC pathways.

## Results

### CNVs in Ulcerative colitis nonprogressor (UC-NP)

We found a relatively small number of copy number variants in the UC-NP group in pooled biopsies from high risk UC patients without any dysplasia. There were 15 CNV regions in total, encompassing 20 genes across different chromosomes (Additional file 1: Table S1). The copy number amplified segment in chromosome 15 was found to be largest harboring 9 genes.

### CNVs in Ulcerative colitis progressor (UC-P)

UC-P samples, comprised of pooled dysplastic and carcinoma biopsy samples, were analyzed against the control samples for aberrant changes happening during the neoplastic changes. A total of 26 chromosomal aberrations were found across the 16 chromosomes listed (Additional file 1: Table S2). More number of gain regions was found across the genome viz., 2q13, 5p13.2, 5q35.3, 5q35.2, 7q31.2-q31.31, 7q32.1, 8p23.1, 8q24.21, 8q24.22, 9p12, 10q23.2, 12p13.33-31, 14q21.1, 15q11.2, 15q13.3, 16p13.11, 16p12.3, 22q11.21, Xq21.31, Yp11.31, as compared to the regions with loss, which were very few and smaller in length and were spread across 3q26.1, 4q13.2, 8p11.23, 11q11, and Xp22.31. About 122 genes were found to be embedded within these CNV altered regions.

### CNVs in Sporadic colorectal cancer (S-CRC)

The S-CRC microarray data highlighted a number of chromosomal regions encompassing protein-coding genes, which exhibited copy number variations (Additional file 1: Table S3). A total of 25 aberrant regions spanning 11 chromosomes, containing more than 400 genes, were observed from the S-CRC sample. Overall gains were observed in 4q34.1, 6p21.32, 8p11.23, 8p12.1-12.3, 8q24.21, 12p13.32, 12p13.31, 13q14.12, 13q21.1, 20p13 and 20q11.1-q13.33. Loss of CNV regions was found across 4p13, 4q13.2, 5p13.2, 5q33.1, 5q35.2, 8p23.1, 8p11.23, 10q23.2, 15q11.2, 22q11.23, Yp11.3, Yp11.2, Yq11.221 and Yq11.223. We observed a large copy number amplified chromosomal segment on chromosome 20q harbouring 381 genes. The smallest region was found in chromosome 4p, consisting of 1000 bp CNV, encompassing a single gene and a ‘gain’ status.

### Integrative data analysis of 244 k arrays in the different groups

**Fig. 1:**
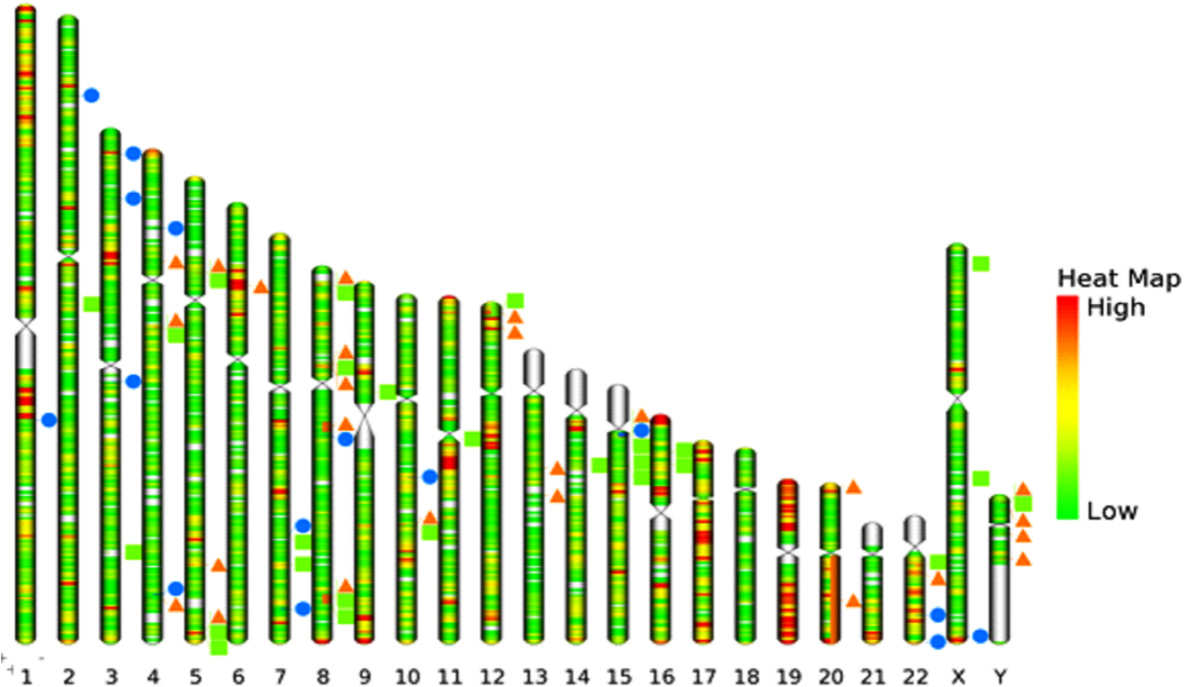
Genome wide chromosomal abnormalities identified in UC-NP (blue circle) samples, UC-P (green square) samples and S-CRC (orange triangle) samples. The heat map is the representative of gene density across each chromosome

A combined analysis of all the three 244 k aCGH microarray data highlighted some of the common and unique CNV regions, and their characteristic behavior in different sample groups (Fig. 1). There were 10 CNV regions across the sample groups, which overlapped with at least one of the sample groups. The chromosome 15 CNV was common to all the three sample groups, with amplification in UC-P and UC-NP, and deletion in S-CRC. Eight CNV regions were common between UC-P and S-CRC, of which 3 regions viz., 4q13.2 (Loss), 8q24.1 (Gain) and 12p13.32 (Gain) showed the same status in both the groups. But the CNV alteration status varied in the other 5 common regions viz., 5p13.2, 5q35, 8p12, 8p23 and 10p regions (Additional file 1: Table S4 and Fig. 1). By using Venny analysis, 9 genes were found to be common between the three groups of samples, while 29 genes were common between UC-P and S-CRC (Additional file 2: Figure S2). A total of 84 out of 122 genes were found to be unique in UC-progressors CNV data and found to have major role in regulating important molecular functions. (Additional file 1: Table S5). Additionally a list of miRNAs identified within the CNV regions is shown in (Additional file 1: Table S6).

### Comparison of CRC genomic profiles of CNV data vs. TCGA data

A comparative analysis was performed between our CNV data and data from The Cancer Genome Atlas Project (TCGA) on sporadic CRC. A number of regions from the TCGA data overlapped with our sample data sets (Additional file 1: Table S7). Eight of the CNV regions from our S-CRC data corresponded to the TCGA reported CNV regions, though the CNV regions found in our study were much smaller in length. The matching CNV status was almost similar except for one small region on chromosome 4 that was reported as a deletion in TCGA data, while we found it to be amplified. In case of UC-P, there were 6 common CNV regions between our data and TCGA regions. Interestingly, a CNV region on chromosome 15 amplified in UC data (both UC-P and UC-NP), was found to be deleted in our S-CRC group and TCGA (CRC) data. However, amplification of CNV regions in chromosomes 8 and 12 was common in all the three data sets (Additional file 1: Table S7).

### Gene set enrichment analysis (GSEA) and Gene Ontology and Pathway Analysis of gene lists from 244 k aCGH data

Genes from the CNV regions obtained from our 244 k aCGH study were stratified on the basis of their known role in cancers using the Broad Institute’s GSEA analysis. The S-CRC data showed 6 oncogenes, 1 tumor suppressor and 36 transcription factors (Additional file 1: Table S8), while in UC-P, there were 5 oncogenes and 10 transcription factors (Additional file 1: Table S9). *MYC* and *CCND2* were the two common genes in UC-P and S-CRC, as highlighted in GSE analysis. We performed a gene ontology search for common biological processes affected by these genes using the DAVID tool. The significant gene ontology terms under biological process of S-CRC and UC-P groups are highlighted (Additional file 2: Figure S3), with cell cycle control being a common term enriched (*p* < 0.05) in these groups. The significant targeting of KEGG pathways agreed well with results showing gene sets from CNVs of both S-CRC and UC-P to target some of the major cancer pathways. CNV genes from UC-P were significantly involved with MAPK and Wnt signaling pathways, whereas S-CRC genes were significantly matched with TGF-beta signaling pathway (Additional file 1: Table S10).

## Quantitative RT-PCR analyses

### MSI and CIN status

The normal, UC-P and UC-NP samples analyzed were microsatellite stable (MSS). In the S-CRN group of samples, 16/98 (16.3 %) samples showed MSI, out of which 4/18 (22.2 %) were in adenomas and 12/80 (15 %) were in adenocarcinomas. Out of 16 MSI positive samples, only 4 did not show any chromosomal instability for the markers analyzed in our qRT-PCR study.

Validation of six genes, *MYC, MYCN, CCND1, CCND2, EGFR* and *FNDC3A* across the three major groups of samples are shown in (Additional file 2: Figure S4, S5 ) and Table 1. C-*MYC* (22.5 %) and *FNDC3A* (20.6 %) were significantly amplified in S-CRN as compared to that of normal samples. In case of UC-HR samples only C-*MYC* (16.1 %) gene was significantly amplified when compared to normal. *FNDC3A* in S-CRN was significantly amplified as compared to both normal and UC-HR samples implying its specificity in sporadic CRC pathway.

**Table 1:** The summary of quantitative real-time PCR results for potential six candidate oncogenes amplification in study group of samples

Gene	S-CRN *n* = 102	UC-CRN *n* = 31	Controls *n* = 30	^1^ *p*-value	^2^ *p*-value
MYC					
Amp n (%)	23 (22.5 %)	5 (16.1 %)	0	*0.004*	*0.05*
Nor n (%)	79 (77.5 %)	26 (83.9 %)	30 (100 %)		
MYCN					
Amp n (%)	16 (15.7 %)	2 (6.5 %)	1(3.3 %)	NS	NS
Nor n (%)	86 (84.3 %)	29 (93.5 %)	29 (96.7 %)		
EGFR					
Amp n (%)	21 (20.6 %)	1 (3.2 %)	2 (6.7 %)	NS	NS
Nor n (%)	81 (79.4 %)	30 (96.8 %)	28 (93.3 %)		
FNDC3A					
Amp n (%)	21 (20.6 %)	2 (6.5 %)	1 (3.3 %)	*0.04*	NS
Nor n (%)	81 (79.4 %)	29 (93.5 %)	29 (96.7 %)		
CCND1					
Amp n (%)	10 (9.8 %)	3 (9.7 %)	0	NS	NS
Nor n (%)	92 (91.2 %)	28 (91.3 %)	30 (100 %)		
CCND2					
Amp n (%)	13(12.7 %)	3 (9.7 %)	1 (3.3 %)	NS	NS
Nor n (%)	89 (87.3 %)	28 (91.3 %)	29 (96.7 %)		

Amp: amplification of gene (copy number >2); Nor: normal gene copy (copy number ≤2);*n* = number of samples;^1^ *p*-value: statistical comparison between S-CRN vs Control; ^2^ *p*-value: statistical comparison between UC-CRN vs Control; NS: statistically not significant (*p* > 0.05). S-CRN group is comprised of 98 samples and 4 cell lines

### Gene to gene interaction, correlation and functional pathway analysis

The associations between the raw copy number score of each sample across all the 6 genes was used to measure the correlation between any two genes (Additional file 2: Figure S4). Raw copy numbers of *EGFR* and *CCND1* was the only positive significant correlation in UC-HR (*r* = 0.430, *p* < 0.05), while with the highest positive correlation in S-CRN group (*r* = 0.372, *p* < 0.01) (Table 2). The 6-gene genomic instability marker panel was designed as a signature that might be involved in important mechanisms of tumor genesis and progression. Towards this, functional pathway analysis for this panel was performed based on database of molecular interactions reported in the literature using Ingenuity Pathway Analysis (IPA) and using cBioPortal, which showed strong interactions between cancer genes and the pathways (Additional file 2: Figure S6).

**Table 2:**
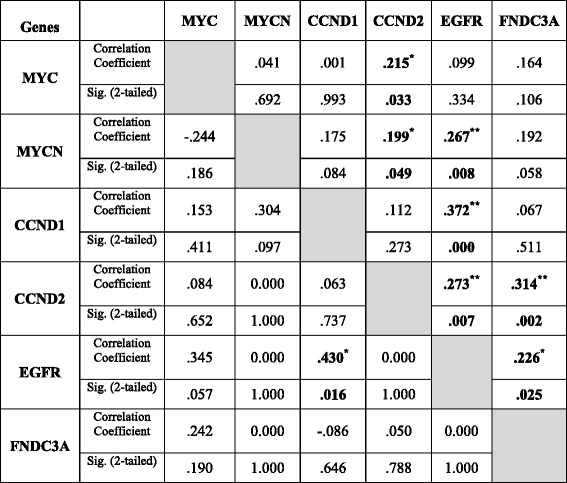
Correlation coefficients of gene copy number between six amplified genes in S-CRN and UC-CRN tissues

Pearson Correlation coefficients and *P*-values were determined as described in the materials and methods. Above the diagonal indicates S-CRN samples tissues (*n* = 98) and below the diagonal indicates UC-CRN samples (*n* =31).*. Correlation is significant at the 0.05 level (2-tailed). **. Correlation is significant at the 0.01 level (2-tailed)

### Sensitivity and specificity for the gene panel

The chromosomal instability signature using the current 6-marker panel was observed in 54/98 (54.1 %) of sporadic colorectal neoplasia patients without MSI. In the same S-CRN group of patients, combination of this panel along with MSI increased the neoplasia detection up to 58.2 %. In case of UC-HR samples, 9/31 (29 %) of the samples showed chromosomal instability (Table 3 and Additional file 1: Table S11).

**Table 3:** Analysis of significance of gene amplification using 6-gene marker panel

	Controls *n* = 30	S-CRN *n* = 98	UC-CRN *n* = 31
0 marker amplified	24 (80 %)	45(45.9 %)	22 (71 %)
≥1 marker amplified	6 (20 %)	53(54.1 %)^*#^	9 (29 %)

*p = 0.001: *p*-value comparing S-CRN with control; ^#^ *p* = 0.02: *p*-value comparing S-CRN with UC-CRN

### Immunohistochemistry analyses

For IHC scoring of p53 and CCND1 proteins, the intensity of nuclear staining was considered. For Ki-67, only the percentage of positively stained nuclei was assessed, as the intensity was similar in all positive nuclei. Membranous staining was assessed for *ERBB2*, whereas for *EGFR* both membranous and cytoplasmic staining was scored. For *C-MYC*, *AMACR* and *FNDC3A* genes only cytoplasmic staining was assessed.

When analyzed together, the 8 markers the typically showed no or weak immunostaining in the nonprogressor tissues, while the immunostaining was frequently moderate to strong in dysplastic or cancerous tissues in UC-HR group (Table 4 and Additional file 2: Figure S7). *p53* and *CCND1* showed significant immunostaining from early high risk stage to neoplastic change. *AMACR* and *EGFR* were more specific for neoplastic changes in both UC-HR and S-CRN. *C-MYC* and *ERBB2* were expressed at lower intensity in both UC-P and S-CRN tissue samples. In the proliferative marker Ki67 expression analysis a significantly higher proliferation index (*p* < 0.05) for both UC-P and S-CRN groups was observed as compared to that of UC-NP (Additional file 2: Figure S8). *FNDC3A*, a cytoplasmic protein, was found strongly overexpressed in all sporadic adenocarcinoma samples. In case of UC-NP and UC-P, 55 % and 88.9 % of the samples respectively showed positive immunostaining for FNDC3A.

**Table 4:** Staining patterns of each immunohistochemical marker in sample groups of non-progressor or with progressors and sporadic colorectal neoplasia

Genes	*UC-NP n = 20*	*UC-P n = 18*	*S-CRN n = 14*	^1^ *p*-value	^2^ *p*-value	^3^ *p*-value
p53 +	*7 (35 %)*	*16 (89 %)*	*9 (64 %)*	*0.0009*	*0.1*	*0.1*
<50 %	2 (28.6 %)	1 (6.2 %)	1 (11.1 %)			
>50 %	5 (71.4 %)	15 (93.8 %)	8 (89 %)			
CCND1 +	*12 (60 %)*	*17 (94 %)*	*14 (100 %)*	*0.02*	*0.01*	NS
<25 %	9 (75 %)	5 (29.4 %)	–			
>25 %	3 (25 %)	12 (70.6 %)	14 (100 %)			
AMACR +	*–*	*9 (50 %)*	*6 (43 %)*	*–*	*–*	*–*
<5 %	–	2 (22.2 %)	–			
>5 %	–	7 (77.8 %)	6 (100 %)			
EGFR +	*–*	*10 (56 %)*	*7 (50 %)*	*–*	*–*	*–*
<5 %	–	2 (20 %)	1 (14.3 %)			
>5 %	–	8 (80 %)	6 (85.7 %)			
C-MYC +	*4 (20 %)*	*10 (56 %)*	*7 (50 %)*	*0.04*	*0.1*	*NS*
<5 %	2 (50 %)	1 (10 %)	2 (28.6 %)			
>5 %	2 (50 %)	9 (90 %)	5 (71.4 %)			
ERBB2 +	*2 (10 %)*	*4 (22 %)*	*6 (43 %)*	*NS*	*0.04*	*NS*
<5 %	2 (100 %)	–	2 (33.3 %)			
>5 %	–	4 (100 %)	4 (66.7 %)			
FNDC3A +	*11 (55 %)*	*16 (88.9 %)*	*14 (100 %)*	*0.032*	*0.004*	*NS*

The number and percentage of patients with a positive immunostaining according to staining intensity and the percentage of stained cells in these patients.^1^ *p*-value: statistical comparison between UC-NP vs UC-P; ^2^ *p*-value: statistical comparison between UC-NP vs S-CRN; ^3^ *p*-value: statistical comparison between UC-P vs S-CRN

## Discussion

From our previous study we observed that CNVs are progressively associated with the development and progression of different stages from UC to CRC [21]. The present study has identified genome-wide altered CNV regions in tissues of UC-progressors, in comparison with S-CRC. An attempt was made to create a panel of markers, including two genes (*C-MYC* and *CCND2*) common to both the pathways, along with other correlated genes, which was evaluated in a larger cohort of either condition for their usefulness in the detection of neoplasia in both CRC conditions. The four noteworthy genes from the above qRT-PCR study were combined complimentarily with four reported markers in CRC and were together analyzed for their expression in a subset of both sporadic and UC neoplasia samples. The current study provides an overview of information on genomic aberrations present in UC associated and sporadic neoplasia and possible markers of importance of disease and molecular pathophysiology. These results can possibly help to better understand the CNVs and the genes involved in the adenoma-carcinoma and dysplasia-carcinoma progression.

The current study is from a region known for its lower prevalence of both UC and CRC, but showing an increasing trend in recent times, although the exact prevalence of these diseases is contentious [22–25]. A recent estimation highlighted an increase of CRC by 2.7 % in developing countries like India [1–3]. But clinical and molecular reports on S-CRN and UC-CRN are scarce from this region. The present study is one of its first types to study integrating aCGH, qRT-PCR and IHC analyses of neoplastic changes in both colitis-associated and sporadic neoplasms for identifying major genomic alterations across the two pathways of CRC development. The bioinformatics-based enrichment analysis along with the comparison with TCGA data showed many overlapping CNVs reinforcing the importance of these altered regions and genes associated with them.

Reports on the use of advanced microarray techniques for UC-CRC are uncommon and studies are lacking on the comparative analysis of CNVs in UC and S-CRC. Using aCGH, the present study has demonstrated important unique and common CNVs associated with neoplasia progression in both UC and sporadic neoplastic pathway. One of the comparative studies by Aust and colleagues [2000] on UC and S-CRC using chromosomal CGH highlighted differences in the frequency and timing of individual alterations suggesting various pathways that operate between the two groups [26]. Earlier studies found that losses in 8p, 15q and 18q and gains in 8q, 13q and 20q were the most common copy number alterations associated with the progression of colorectal adenoma to carcinoma [26–30]. In the current analysis, we found 13q and 20q amplifications in S-CRC alone, but 8q amplifications were present in both UC-P and S-CRC samples. In comparison with S-CRC data, UC-P had noticeably smaller CNV regions with more gain statuses (for example, in chromosomes 7, 8, 12 and 22). Interestingly 15q CNV was one of the common CNVs between the 3 sample groups amplified in UC samples, but deleted in S-CRC. Common CNV regions and genes emerged from integrated analysis of UC-P and S-CRC suggests a common molecular function is regulated in neoplastic epithelial cells. The chromosomal 8q and 12p regions comprises of important functional genes such as *C-MYC* and *CCND2* oncogenes and may drive sporadic as well as inflammation associated carcinogenesis. Bioinformatics analysis and other studies too have highlighted the importance of these CNVs and genes [11, 31]. Thus, these results may help broaden our understanding of the inter-related molecular pathways in the two conditions.

Studies on whole genome aberrations have been attempted to identify and test potential markers for translation, since few markers are currently being recommended for use in the clinical practice [32]. The cancer genome atlas project (TCGA) is among the major initiative in this aspect and has reported a comprehensive genome-scale analysis of genetic variations across 276 CRC samples [29]. The overlapping analysis of our aCGH based CNV results with TCGA data has shown many similar CNV regions and these CNVs can be tested across populations.

Much effort has also been devoted to the development of panel of markers based on genetic and epigenetic alterations in different cancers [33, 34]. We attempted to establish a panel of markers from the CNV regions and validated the same in our patient’s cohort using qRT-PCR. Towards this effort, a 6-gene genomic instability signature for neoplastic changes was designed and validated in both the colorectal cancer types. The 3 genes (C-MYC, CCND2 and FNDC3A) were selected from our data and together with the previously published genes (*MYCN, CCND1* and *EGFR*), we generated a panel of 6 genes for validation. Functional pathway enrichment analysis was carried out based on curated database using Ingenuity Pathway Analysis and cBioPortal using TCGA-CRC data. The current panel, considering alterations in at least one marker, was efficient in detecting neoplastic changes in more than 50 % of the samples in S-CRC but was comparatively less in UC-neoplastic samples. Combination of MSI and qRT-PCR panel did not significantly improve the sensitivity of detection. In correlation analysis, we found that *EGFR* and *CCND1* raw copy number values are positively correlated with neoplastic changes in both UC and S-CRN samples.

There are several reports on the gene amplifications in CRC that has been correlated to gene expression [13, 14, 35–37]. We tested by IHC using 8 markers which is a combination of previously reported markers and from our qRT-PCR study. Results conclude that *p53*, *CCND1*, *EGFR*, *C-MYC* and *FNDC3A* were overexpressed more than 50 % of the time in S-CRN samples. Interestingly in UC-HR samples, it was observed that *p53* and *CCND1* were significantly expressed at higher frequency compared to tissues from preneoplastic stages, while C-*MYC* and *ERBB2* were expressed at very low frequency. *EGFR* and *AMACR* expression was more specific towards neoplastic changes and showed a linear relationship with increasing disease frequency.

Fibronectin type III domain containing 3A (*FNDC3A*) gene is shown to be involved in major biological function of cell-cell adhesion and is one of the genes from the widely reported 13q CNV region in S-CRC. However, very little is known about the role of this gene in cancer. FNDC3A gene showed amplified copy number status in both aCGH and qRT-PCR, and overexpressed in tissue samples of S-CRC. The functional significance of *FNDC3A* warrants further study in adenocarcinoma. In accordance with our previous findings on p53 mutational analysis, to the current IHC results suggest that the p53 pathway is perhaps an early event and Wnt-pathway regulated changes in *C-MYC* are in the later phase of colitis associated carcinogenesis [38]. In clinical practice, assessment of the expression of these markers may help to identify patients with risk of neoplasia, thereby supporting the surveillance strategies and therapy.

Pooled sample-based analysis has been recognized as a cost-effective alternative approach for filtering genetic variance of higher significance, though chances of missing less frequent CNVs exist [39, 40]. The success of sample pooling based arrays depends upon reducing the overall pooling error however, errors due to array specific variability remains. The important and major CNV regions (e.g. 8q, 13q, 20q amplifications) reported in this study across the CRC genome have been retained even after the pooling. Sampling biases due to tissue heterogeneity and multifocality of epithelium have been the limiting factors in CRC molecular analysis [40]. MSI and CIN analysis by qRT-PCR could have been affected by these above factors. Another limitation of these assays is that their detection thresholds usually need clonal expansion and broad field effects of the targeted cell population being tested [41]. The number of patients in each group was relatively low, which requires a careful interpretation of the results. Similarly in the IHC study, the degree of immunoreactivity of each antibody may frequently heterogeneously distributed throughout the tissue sample [42]. To avoid selection bias during the scoring, we selected the area with the strongest immunoreactivity in each tissue sample [42, 43]. In order to predict the prognosis and therapeutic outcome, series of studies have established biomarker panels for S-CRC. However, consensus on the suitable biomarkers for early diagnosis remains to be established [14, 44]. In the current study, we have attempted to simultaneously analyze two CRC related using panel of markers to aid in further understanding of molecular pathogenesis. The study has integrated some of the well-known marker genes along with the relatively new loci from the current study in the analysis as a group and highlighted their importance in early phases of cancer development and detection. These may help in understanding and targeting the different stages of CRC development in UC patients who are on continuous follow-up for their disease evaluation. The surveillance program remains cumbersome and addition of these markers along with clinical follow up to increase the efficiency of neoplasia detection can lead to better and successful screening strategies. Of significance is that this is the only report from India and among a very few elsewhere, to have comparatively analyzed and validated CNVs and the genes together and the expression patterns of markers in both UC and sporadic colorectal neoplasia.

## Conclusion

Our aCGH analysis demonstrated that colitis associated and sporadic colorectal carcinomas do contain a varied level of CIN in the form of CNVs and are common to CRC pathways. Overlapping of our data with TCGA-CRC data indicated common CNVs across the populations. The marker panel based validation study by using qRT-PCR and IHC may help to delineate choice of markers from CNV regions for identification of CRC. Reproducibility testing with a larger cohort and longitudinal analyses over time is required to assess the role of CNVs as potential markers. Comparative CNV analysis on colitis associated and sporadic cancer genomes has provided the testable loci for possible aberrant driver events. Using advanced colonoscopic techniques to target the abnormal areas for neoplasia detection followed by targeted molecular analysis may help in screening and follow up programs towards effective treatment strategies.

## Methods

### Experimental design

Study was approved by the Kasturba Hospital Ethics Committee (KHEC No.159/07), Manipal. All the patients provided written informed consent before participation. Tissue samples were obtained from biopsy of the patients, further divided into following groups UC-nonprogressors (UC-NP): 20 UC patients with high risk but without any dysplasia, UC-progressors (UC-P): 08 patients with dysplasia or cancer, and sporadic colorectal cancer (S-CRC): 20 patients. A pool of DNA from 20 (10 male and 10 female) endoscopically and histopathologically normal colon were used as the control samples for all the arrays. For all DNA based assays, DNA was isolated from the tissue using phenol-chloroform method. To search for genetic variations, the experimental design comprised of the hybridization of tissue DNA samples from above mentioned groups of patients against a control pool consisting of the non-tumor tissue.

For validation by qRT-PCR study, UC-HR group comprised of thirty-one patients with UC at risk of associated colorectal neoplasia (≥7 years of extensive colitis or ≥10 years of left-sided colitis) were included in the analysis. These samples were further classified as UC progressor (*n* = 14) and UC non-progressor (*n* = 17) based on the presence or absence, respectively, of neoplastic changes. The sporadic colorectal neoplasia samples were collected through colonoscopy from 98 patients, of whom 80 were adenocarcinomas and 18 were adenomas. The control group consisted of DNA extracted from 15 men and 15 women subjects with no organic colonic disease (Colonoscopicaly and histopathologically confirmed) (Table 5).

**Table 5:** Clinical details of the samples in the quantitative real-time PCR validation study

	Sample	N	Male:Female	Median Age (range) in years
Controls	Normal	30	15:15	52.5 (18–72)
Sporadic Colorectal neoplasia (S-CRN)	Adenoma	18	16:02	56.5 (19–79)
	Adenocarcinoma	80	51:29	55 (07–88)
Ulcerative colitis associated colorectal neoplasia (UC-CRN)	UC-Nonprogressor	17	10:07	54 (18–68)
	UC-Progressor	14	08:06	49.5 (20–69)

For IHC-based expression analysis in UC-HR, group comprised of 38 samples. Out of these 18 were progressor and among these 18 samples LGD was found in 5, HGD in 9 and UC associated CRC in 4 samples. The comparative S-CRN group comprised of 14 patient samples out of which 4 were primary colorectal cancer and 10 adenoma samples. For IHC experiment, each sample was confirmed with initial Hematoxylin and Eosin (H&E) grading.

Those with S-CRN underwent endoscopic biopsies from affected and normal areas for histology and molecular analysis. The diagnosis of both UC and CRN was made according to established criteria, including clinical symptoms, colonoscopy and histopathology. Human colorectal cancer cell lines CACO-2, COLO-205, HT-29 and HCT-15 were obtained from National Centre for Cell Science (NCCS, India) and DNA extracted from them was used in the initial analysis. The overall study design has been elucidated in (Additional file 2: Figure S1). Briefly, to identify of genome wide CNVs contributing to both UC associated neoplasia and sporadic CRC development, we performed 244 k aCGH experiment. The aCGH results were analysed for common and unique CNVs to both the samples and enrichment of CNVs for functional annotation using bioinformatics tools that overlap with TCGA data and literature was performed. Three genes (*C-MYC, CCND2* and *FNDC3A*) were selected from our data and together with previously reported (*MYCN, CCND1* and *EGFR*) genes were validated using Taqman CNV based qRT-PCR assay on UC-high risk, sporadic colorectal neoplasia and compared against control samples. Subsequently, the four genes (*C-MYC, CCND1, EGFR* and *FNDC3A*) from the above qRT-PCR study were assessed along with four previously reported markers (p53, AMACR, ERBB2 and Ki67) for their expression by IHC in both UC and sporadic CRC sample.

### Microarray platform

aCGH was performed using the Agilent Human Genome Microarray Kit (Agilent Technologies, Santa Clara, CA) microarrays. This array contained 236,381 distinct biological 60-mer oligonucleotide probes, with 1,000 biological triplicates and 5,045 controls spanning coding and non-coding genomic sequences with median probe spacing of 7.4 and 16.5 kb, respectively. The average probe spacing was 6.4 kb was calculated by dividing total repeat-masked genome size by total microarray features. The probe sequences and gene annotations were based on NCBI Build 36.1 of the human genome and UCSC version hg18 released in May 2006.

### Microarray analysis

Copy number variation (CNV) analysis of UC-nonprogressor, UC-progressor and sporadic CRC was performed using Agilent high-density 244 K microarray. Briefly, DNA samples were sheared using a cycle of 15 s ‘on’ and 15 s ‘off’ for 15 min in an ultrasonic processor (Thomas Scientific, NJ, USA) with a 2 mm probe with amplitude set at 40. The purified sheared DNA was differentially labeled, test samples DNA (test genome) with fluorescent Cy5 and the pooled normal reference (control genome) DNA with Cy3 dyes. Hybridization, washing and scanning of the arrays were performed according to the manufacturer’s protocol. Feature extracted data was analyzed with Genomic Workbench v5.0 software (Agilent Technologies, CA, USA) using ADM-2 aberration detection algorithm (threshold 5.0) and visual inspection of the log2 ratios (±0.25) [45]. Gene enrichment, gene ontology and pathway analysis were carried out using GSEA, DAVID, PANTHER, cBioPortal and KEGG bioinformatics tools.

### Multiplex PCR based Microsatellite Instability (MSI) Analyses

Microsatellite instability (MSI) status was examined using 5 microsatellite markers (National Cancer Institute, Bethesda Panel). The assay was carried out using the primer sequences and the corresponding fluorescent dyes and PCR as described elsewhere [46]. In brief, multiplex PCR was performed in a Veriti thermocycler (Applied Biosystems, Foster City, CA) using the following cycling conditions: 95 °C for 2 min, followed by 30 cycles of 94 °C for 30 s, 55 °C for 30 s and 72 °C for 30 s, with a final 45 min, 60 °C extension to aid non-template adenine addition. The PCR products were analyzed using ABI 3130 Genetic Analyzer (Applied Biosystems, Foster City, CA) along with GS500LIZ size standard according to the manufacturer’s instructions. The generated data were analyzed using Genemapper v.4.0 (Applied Biosystems, Foster City, CA). If there was a peak shift or presence of abnormal alleles at zero, one or more microsatellite loci tested compared with the normal control DNA from the same patient, the samples were graded as microsatellite stable (MSS) or microsatellite instable (MSI) respectively.

### Copy number determination by quantitative real-time PCR (qRT-PCR)

The number of copies of C-*MYC*, *CCND2* and *FNDC3A* genes from our data were combined with their correlated interacting partners *MYCN*, *CCND1* and *EGFR* genes (these genes were found to be within the cut off log2 ratios in our aCGH data) in tumor cell lines and tumor tissue samples from cancer patients was determined by quantitative real time polymerase chain reaction (qRT-PCR). TaqMan® copy number assays (Applied Biosystems, Foster City, CA) were applied and the details of the genes are listed in (Additional file 1: Table S12). These assays were performed on the 7500 Fast Real Time PCR system with Sequence Detection System v2.4 (Applied Biosystems, Foster City, CA, USA) software. Amplification reaction mixtures (10 ul) for each target gene contained template DNA (10 ng), final 1x concentration of TaqMan® universal master mix, TaqMan® copy number assay reagent, and TaqMan® copy number reference assay (RNAseP) in a 96-well plate. The cycling conditions used were 10 min at 95 °C, followed by 40 cycles of 15 s at 95 °C and 60 s at 60 °C. After running each experiment in triplicates, data files containing the sample replicate Ct values for each reporter dye were exported from the real-time PCR instrument software into Copy Caller software v.1, which calculates each sample copy number values based on relative quantitation (comparative Ct method).

### Immunohistochemistry (IHC) analysis

The four noteworthy genes *C-MYC, CCND1, EGFR* and *FNDC3A* from the above qRT-PCR study were combined with four reported markers *p53*, *AMACR*, *ERBB2* and *Ki67* (these genes were found to be within the cut off log2 ratios in our aCGH data) in CRC (Additional file 1: Table S13). Sections (5–7 micron thick) from formalin-fixed, paraffin-embedded tissue samples were applied to poly-L-lysine coated slides. The sections were dewaxed in xylene and rehydrated and an antigen retrieval step was done. After antigen retrieval by microwaving, immunostaining was performed using the biotin–streptavidin–peroxidase method. Counterstaining was carried out with hematoxylin. Immunostaining for all the antibodies was assessed according to the intensity of staining and divided into four categories: negative (-), weak (+), moderate (++), or strong (+++), with moderate or strong IHC staining being regarded as positive. For staining frequency of these antibodies, the number of positive (moderate or strong) cells were expressed as the percentage of the total number of cells per high-power field and categorized as 5 %–25 %, 25 %–50 %, 50 %–75 %, and >75 %.

### Statistical analysis

Statistical significance was defined by *P*-values of ≤ 0.05. Correlations between copy numbers of the six amplified genes were calculated using Spearman’s rank correlation coefficient (r). Expression patterns of the individual IHC markers were compared between patients with progression to advanced neoplasia and those without progression and other subgroups using Fisher’s exact test or chi-square test, as appropriate. Statistical analyses were carried out using SPSS 15.0 (IBM) and GraphPAD InStat (California, USA) software.

## Additional files

Additional file 1: Table S1. CNVs found in UC-nonprogressor group (UC-NP) group 244 k genomic aberration report. **Table S2.** CNVs found in UC-progressor (UC-P) group 244 k genomic aberration report. **Table S3.** CNVs found in S-CRC group 244 k genomic aberration report. **Table S4.** List and details of the overlapping CNV regions between the three of the study sample groups (S-CRC, UC-P and UC-NP). **Table S5.** List of unique in UC-progressors genes from CNV data. **Table S6.** List of miRNAs overlapping to uniquely shared CNV regions of different sample groups. **Table S7.** Comparative analysis of all CNVs observed in different sub-groups with the data from The Cancer Genome Atlas Network (TCGA) project for CRC. **Table S8.** Gene set enrichment analysis (GSEA) for S-CRC group CNV associated genes. **Table S9.** Gene set enrichment analysis (GSEA) for UC-P group CNV associated genes. **Table S10.** The functional KEGG pathways enriched with genes located on the chromosomal segments with copy number alterations in S-CRC and UC-P samples. **Table S11.** Prediction accuracy of colorectal neoplasia using the 6-gene panel instability signature along with MSI. **Table S12.** Details of TaqMan CNV assays used in the microarray validation study. **Table S13.** Details of antibodies and staining conditions used for Immunohistochemistry (IHC). (PDF 398 kb) Additional file 2: Figure S1. Overall workflow and design of the study. **Figure S2.** Common genes found associated with CNVs in UC non-progressor, UC progressor and sporadic colorectal cancers. **Figure S3.** Enrichment in biological process (GO analysis) of the gene from S-CRC and UC-P samples 244 k aCGH data. X-axis: number of genes involved in the given function and Y-axis: biological function the genes are involved. **Figure S4.** Results from the screening of gene CNVs (amplification and deletion) in subgroups of sporadic and ulcerative colorectal neoplasm samples in our validation panel of markers by qRT-PCR method. (Abbreviations used are as given earlier). **Figure S5.** Clustering of qRT-PCR data using 6 genes and 163 samples of different groups. The relative copy number for each gene was plotted against different sample groups from the current study. **Figure S6.** Summary of Ingenuity Pathways Analysis (IPA) for the role and interaction of the 6 genes markers panel. **Figure S7.** Results of immunohistochemistry analysis carried out on UC associated and sporadic colorectal cancer samples for various proteins; representative images are for **A:** p53 **B:** Cyclin D1; **C:** AMACR; **D:** EGFR; **E:** C-MYC; **F:** ERBB2; **G:** Ki67; **H:** FNDC3A. **Figure S8.** Box plot illustrating percentage of Ki-67 positive cells in different sample groups of the current study: ulcerative colitis-non progressor (UC-NP) group, ulcerative colitis- progressor (UC-P) group and sporadic colorectal neoplasia (S-CRN) group. (PDF 551 kb)

## Abbreviations

aCGH: array comparative genome hybridization
CNVs: copy number variations
IHC: immunohistochemistry
MSI: microsatellite instability
S-CRC: sporadic colorectal cancer
S-CRN: sporadic colorectal neoplasia
S-PM: sporadic–premalignant
UC-CRC or CAC: ulcerative colitis associated colorectal cancer
UC-HR: ulcerative colitis high risk
UC-NP: ulcerative colitis–nonprogressor
UC-P: ulcerative colitis–progressor
