# Supplementary material for: Comparative analysis of copy number variations in ulcerative colitis associated and sporadic colorectal neoplasia
Source: BMC Cancer. 2016 Apr 14;16:271. doi: 10.1186/s12885-016-2303-4 (PMC4831153; doi:10.1186/s12885-016-2303-4)
Supplement: Additional file 1: Table S1. — CNVs found in UC-nonprogressor group (UC-NP) group 244 k genomic aberration report. Table S2. CNVs found in UC-progressor (UC-P) group 244 k genomic aberration report. Table S3. CNVs found in S-CRC group 244 k genomic aberration report. Table S4. List and details of the overlapping CNV regions between the three of the study sample groups (S-CRC, UC-P and UC-NP). Table S5. List of unique in UC-progressors genes from CNV data. Table S6. List of miRNAs overlapping to uniquely shared CNV regions of different sample groups. Table S7. Comparative analysis of all CNVs observed in different sub-groups with the data from The Cancer Genome Atlas Network (TCGA) project for CRC. Table S8. Gene set enrichment analysis (GSEA) for S-CRC group CNV associated genes. Table S9. Gene set enrichment analysis (GSEA) for UC-P group CNV associated genes. Table S10. The functional KEGG pathways enriched with genes located on the chromosomal segments with copy number alterations in S-CRC and UC-P samples. Table S11. Prediction accuracy of colorectal neoplasia using the 6-gene panel instability signature along with MSI. Table S12. Details of TaqMan CNV assays used in the microarray validation study. Table S13. Details of antibodies and staining conditions used for Immunohistochemistry (IHC). (PDF 398 kb) [file 12885_2016_2303_MOESM1_ESM.pdf]

Additional File 2: Table S1 to S13

Table S1: CNVs found in UC-nonprogressor group (UC-NP) group 244k genomic aberration report

Sl. No	Chr	Start	Stop	Size (in bp)	No of Probes	CNV status	No of Genes
**1**	chr1	160806847	160813371	6524	2	Gain	1
**2**	chr2	31201435	31212189	10754	2	Loss	1
**3**	chr3	10016304	10022512	6208	2	Loss	0
**4**	chr3	27468786	27475123	6337	2	Loss	1
**5**	chr3	98066674	98073706	7032	2	Gain	1
**6**	chr4	30883071	30920449	37378	2	Gain	0
**7**	chr4	171984321	172084742	100421	4	Gain	0
**8**	chr7	116078828	116093617	14789	2	Gain	0
**9**	chr7	145068068	145102088	34020	2	Loss	0
**10**	chr8	65703020	65709344	6324	2	Loss	1
**11**	chr10	70775982	70789507	13525	2	Gain	3
**12**	chr15	18741516	20060261	1318745	66	Gain	9
**13**	chr22	38482482	38491719	9237	2	Gain	1
**14**	chr22	48806960	48814822	7862	2	Gain	1
**15**	chrX	151773936	151781641	7705	2	Loss	1

Table S2: CNVs found in UC-progressor (UC-P) group 244k genomic aberration report

Sl. No	Chr	Start	Stop	Size (in bp)	No of Probes	CNV status	No of Genes
**1**	chr2	111891328	111922721	31393	2	Gain	1
**2**	chr3	163997028	164101976	104948	8	Loss	0
**3**	chr4	69138837	69166014	27177	4	Loss	0
**4**	chr5	37491262	37501689	10427	2	Gain	1
**5**	chr5	175590532	175600782	10250	2	Gain	1
**6**	chr5	176992330	177069037	76707	3	Gain	1
**7**	chr7	115891901	117270069	1378168	153	Gain	13
**8**	chr7	127989138	128035933	46795	2	Gain	0
**9**	chr8	7729111	7747972	18861	2	Gain	4
**10**	chr8	39356395	39488481	132086	17	Loss	2
**11**	chr8	127984937	128919983	935046	54	Gain	5
**12**	chr8	130618090	131535168	917078	98	Gain	4
**13**	chr9	42362428	42903250	540822	2	Gain	6
**14**	chr10	89178788	89199963	21175	2	Gain	0
**15**	chr11	55124530	55179155	54625	9	Loss	3
**16**	chr12	33654	5974091	5940437	615	Gain	45
**17**	chr14	37132224	37132624	1000	1	Gain	1
**18**	chr15	18657188	20250086	1592898	72	Gain	9
**19**	chr15	30422992	30649059	226067	2	Gain	2
**20**	chr15	30710135	30710535	1000	1	Gain	1
**21**	chr16	15091992	15159468	67476	3	Gain	1
**22**	chr16	18599299	18687616	88317	2	Gain	0
**23**	chr22	19069125	19776005	706880	106	Gain	19
**24**	chrX	7542346	8091951	549605	37	Loss	2
**25**	chrX	90924906	90933499	8593	2	Gain	1
**26**	chrY	2985364	3035107	49743	3	Gain	0

Table S3: CNVs found in S-CRC group 244k genomic aberration report

Sl. No	Chr	Start	Stop	Size (in bp)	No of Probes	CNV Status	No of Genes
**1**	chr4	43604505	43615473	10968	2	Loss	0
**2**	chr4	68905470	69166014	260555	12	Loss	3
**3**	chr4	173665872	173666872	1000	2	Gain	1
**4**	chr5	37491262	37501689	10427	2	Loss	1
**5**	chr5	150184990	150201345	16355	2	Loss	0
**6**	chr5	175590532	175600782	10250	2	Loss	1
**7**	chr6	32567182	32660290	93108	9	Gain	3
**8**	chr8	7358840	7747972	389132	4	Loss	11
**9**	chr8	39080685	39094953	14268	2	Loss	2
**10**	chr8	39356395	39535855	179460	22	Gain	2
**11**	chr8	60283407	64086021	3802614	281	Gain	7
**12**	chr8	126843399	130486270	3642871	193	Gain	6
**13**	chr10	89178788	89241427	62639	3	Loss	0
**14**	chr12	3455290	5026118	1570828	155	Gain	16
**15**	chr12	9528390	9585356	56966	3	Gain	0
**16**	chr13	48446041	48639147	193106	22	Gain	0
**17**	chr13	56645352	56726208	80856	7	Gain	0
**18**	chr15	18741516	20220616	1479100	68	Loss	9
**19**	chr20	1516766	1539355	22589	3	Gain	2
**20**	chr20	28265913	62379259	34113346	3101	Gain	381
**21**	chr22	22677759	22725505	47746	10	Loss	3
**22**	chrY	2971061	3035107	64046	4	Loss	0
**23**	chrY	5633876	5672641	38765	2	Loss	1
**24**	chrY	15200439	15241847	41408	6	Loss	1
**25**	chrY	24685122	24754407	69285	2	Loss	2

Table S4: List and details of the overlapping CNV regions between the three of the study sample groups (S-CRC, UC-P and UC-NP)

Chr	Start	Stop	Size in bp	No of Probes	Gain	Loss	Average Overlap Fraction (Probes)	Sample names	No of Genes
**chr4**	68905470	69166014	260544	12	0	2	0.077	UC-P and S-CRC	3
**chr5**	37491262	37501689	10427	2	1	1	0.5	UC-P and S-CRC	1
**chr5**	175590532	175600782	10250	2	1	1	0.5	UC-P and S-CRC	1
**chr7**	115891901	117270069	1378168	153	2	0	0.0065	UC-NP and UC-P	15
**chr8**	7358840	7747972	389132	4	1	1	0.2	UC-P and S-CRC	12
**chr8**	39356395	39535855	179460	22	1	1	0.047	UC-P and S-CRC	2
**chr8**	126843399	130486270	3642871	193	2	0	0.0051	UC-P and S-CRC	6
**chr10**	89178788	89241427	62639	3	1	1	0.33	UC-P and S-CRC	0
**chr12**	33654	5974091	5940437	615	2	0	0.0016	UC-P and S-CRC	54
**chr15**	18657188	20250086	1592898	72	2	1	0.014	UC-NP , UC-P and S-CRC	9

Table S5: List of unique genes found in UC-progressor CNV data

Gene Name	Gene ID	Chromosomal Location	Full Name	Protien Family
**SNAP29**	**9342**	22q11.21	synaptosomal-associated protein, 29kDa	SNARE protein
**RRN3**	**54700**	16p12	RRN3 RNA polymerase I transcription factor homolog (S. cerevisiae)	transcription factor
**PI4KA**	**5297**	22q11.21	phosphatidylinositol 4-kinase, catalytic, alpha	kinase
**CACNA2D4**	**93589**	12p13.33	calcium channel, voltage-dependent, alpha 2/delta subunit 4	voltage-dependent calcium channel complex
**NRIP2**	**83714**	12p13.33	nuclear receptor interacting protein 2	transcription cofactor
**RAD52**	**5893**	12p13-p12.2	RAD52 homolog (S. cerevisiae)	DNA binding protein
**OR4P4**	**81300**	11q12.1	olfactory receptor, family 4, subfamily P, member 4	-
**ADIPOR2**	**79602**	12p13.31	adiponectin receptor 2	receptor
**PNPLA4**	**8228**	Xp22.3	patatin-like phospholipase domain containing 4	acyltransferase
**NTF3**	**4908**	12p13	neurotrophin 3	neurotrophic factor
**FOXD4L4**	**349334**	9q21.11	forkhead box D4-like 4	transcription factor
**CACNA1C**	**775**	12p13.3	calcium channel, voltage-dependent, L type, alpha 1C subunit	voltage-gated calcium channel
**OR4C11**	**219429**	11q11	olfactory receptor, family 4, subfamily C, member 11	-
**THAP7**	**80764**	22q11.2	THAP domain containing 7	nucleic acid binding
**SERPIND1**	**3053**	22q11.21	serpin peptidase inhibitor, clade D (heparin cofactor), member 1	serine protease inhibitor
**LRTM2**	**654429**	12p13.33	leucine-rich repeats and transmembrane domains 2	-
**TMEM191A**	**84222**	22q11.21	transmembrane protein 191A (pseudogene)	-
**ITFG2**	**55846**	12p13.33	integrin alpha FG-GAP repeat containing 2	-
**AQP7P3**	**441432**	9p12	aquaporin 7 pseudogene 3	transporter
**ARHGAP11A**	**9824**	15q13.2	Rho GTPase activating protein 11A	G-protein modulator
**ZNF74**	**7625**	22q11.21	zinc finger protein 74	-
**FOXD4L2**	**100036519**	9q21.11	forkhead box D4-like 2	transcription factor
**WNT5B**	**81029**	12p13.3	wingless-type MMTV integration site family, member 5B	signaling molecule
**PCDH11X**	**27328**	Xq21.3	protocadherin 11 X-linked	cadherin
**LZTR1**	**8216**	22q11.1-q11.2	leucine-zipper-like transcription regulator 1	-
**POM121L4P**	**266697**	22q11.2	POM121 transmembrane nucleoporin-like 4 pseudogene	
**ERC1**	**23085**	12p13.3	ELKS/RAB6-interacting/CAST family member 1	membrane traffic protein
**CCDC77**	**84318**	12p13.33	coiled-coil domain containing 77	-
**VCX**	**26609**	Xp22	variable charge, X-linked	-
**SLC7A4**	**6545**	22q11.21	solute carrier family 7 (orphan transporter), member 4	amino acid transporter
**P2RX6**	**9127**	22q11.21	purinergic receptor P2X, ligand-gated ion channel, 6	ligand-gated ion channel
**CFTR**	**1080**	7q31.2	cystic fibrosis transmembrane conductance regulator (ATP-binding cassette sub-family C, member 7)	anion channel
**ASAP1**	**50807**	8q24.1-q24.2	ArfGAP with SH3 domain, ankyrin repeat and PH domain 1	nucleic acid binding
**WNK1**	**65125**	12p13.3	WNK lysine deficient protein kinase 1	non-receptor serine/threonine protein kinase
**FKBP4**	**2288**	12p13.33	FK506 binding protein 4, 59kDa	isomerase
**P2RX6P**	**440799**	22q11.21	purinergic receptor P2X, ligand-gated ion channel, 6 pseudogene	
**AIFM3**	**150209**	22q11.21	apoptosis-inducing factor, mitochondrion-associated, 3	dehydrogenase
**KLHL22**	**84861**	22q11.21	kelch-like 22 (Drosophila)	-
**FAM49B**	**51571**	8q24.21	family with sequence similarity 49, member B	-
**OR4S2**	**219431**	11q11	olfactory receptor, family 4, subfamily S, member 2	-
**IQSEC3**	**440073**	12p13.33	IQ motif and Sec7 domain 3	guanyl-nucleotide exchange factor
**CAV2**	**858**	7q31.1	caveolin 2	transmembrane receptor regulatory/adaptor protein
**ST7**	**7982**	7q31.2	suppression of tumorigenicity 7	-

**SCARF2**	**91179**	22q11	scavenger receptor class F, member 2	receptor
**CTTNBP2**	**83992**	7q31	cortactin binding protein 2	non-receptor serine/threonine protein kinase
**MED15**	**51586**	22q11.2	mediator complex subunit 15	-
**SLC6A13**	**6540**	12p13.3	solute carrier family 6 (neurotransmitter transporter, GABA), member 13	cation transporter
**MET**	**4233**	7q31	met proto-oncogene (hepatocyte growth factor receptor)	-
**GSDMC**	**56169**	8q24.21	gasdermin C	-
**LOC202181***	**202181**	5q35.3	chromosome 5 open reading frame 25 pseudogene (SUMO-interacting motifs containing 1 pseudogene)	
**VWF**	**7450**	12p13.3	von Willebrand factor	extracellular matrix glycoprotein
**CAV1**	**857**	7q31.1	caveolin 1, caveolae protein, 22kDa	transmembrane receptor regulatory/adaptor protein
**CAPZA2**	**830**	7q31.2-q31.3	capping protein (actin filament) muscle Z-line, alpha 2	non-motor actin binding protein
**B4GALNT3**	**283358**	12p13.33	beta-1,4-N-acetyl-galactosaminyl transferase 3	glycosyltransferase
**DCP1B**	**196513**	12p13.33	DCP1 decapping enzyme homolog B (S. cerevisiae)	transcription cofactor
**TEAD4**	**7004**	12p13.3-p13.2	TEA domain family member 4	transcription factor
**LOC541471***	**541471**		uncharacterized LOC541471	
**NINJ2**	**4815**	12p13	ninjurin 2	cell adhesion molecule
**TULP3**	**7289**	12p13.3	tubby like protein 3	-
**FOXM1**	**2305**	12p13	forkhead box M1	transcription factor
**ANKRD20A3**	**441425**	9p12	ankyrin repeat domain 20 family, member A3	-
**FBXL14**	**144699**	12p13.33	F-box and leucine-rich repeat protein 14	-
**FAM95B1***	**100133036**	9p12	family with sequence similarity 95, member B1	-
**SLC6A12**	**6539**	12p13	solute carrier family 6 (neurotransmitter transporter, betaine/GABA), member 12	cation transporter
**ANKRD20A2**	**441430**	9p12	ankyrin repeat domain 20 family, member A2	-
**WNT2**	**7472**	7q31.2	wingless-type MMTV integration site family member 2	signaling molecule
**KDM5A**	**5927**	12p11	lysine (K)-specific demethylase 5A	zinc finger transcription factor
**ANO2**	**57101**	12p13.3	anoctamin 2	Calcium Activated Chloride Channel
**FOXA1**	**3169**	14q21.1	forkhead box A1	transcription factor
**TSPAN9**	**10867**	12p13.33-p13.32	tetraspanin 9	membrane-bound signaling molecule
**CRKL**	**1399**	22q11.21	v-crk sarcoma virus CT10 oncogene homolog (avian)-like	activate the RAS and JUN kinase signaling pathways
**ASZ1**	**136991**	7q31.2	Ankyrin Repeat, SAM And Basic Leucine Zipper Domain Containing 1	signal transducer activity
**ST7OT1***	**93653**	7q31.2	ncRNA	-
**C12orf32**	**83695**	12p13.33	RAD9-HUS1-RAD1 interacting nuclear orphan 1	DNA damage response (DDR) signaling
**FAM7A1***	**89838**	15q13.3	ULK4 pseudogene 1	-
**ST7OT3**	**93655**	7q31.3	ST7 overlapping transcript 3	-
**ST7OT2***	**93654**	7q31.2	ncRNA	-
**HSN2**	**NULL**	Alternate name for WNK1	WNK lysine deficient protein kinase 1	WNK subfamily of serine/threonine protein kinases
**ST7OT4***	**338069**	7q31.2	ncRNA	-
**DDEF1IT1***	**29065**	8q24.21	ncRNA	-
**FAM7A2***	**100288380**	15q13.2	ULK4 pseudogene 2	-
**MGC16703***	**113691**	22q11.21	tubulin, alpha 3f, pseudogene	-
**LRRC74B**	**400891**	22q11.21	leucine rich repeat containing 74B	laminin binding
**FLJ39582***	**439931**	22q11.21	Uncharacterized Protein FLJ39582-Like	RNA Gene

*nc RNA, Pseudogenes or experimental level data for proteins is not available

Table S6: List of miRNAs overlapping to uniquely shared CNV regions of different sample groups

Chr	Sample	miRNAs
**Chr 8**	S-CRC and UC-P*	hsa-mir-1204* , hsa-mir-1205 , hsa-mir-1206 , hsa-mir-1207 , hsa-mir-1208
**Chr 15**	S-CRC and UC-P	hsa-mir-1268
**Chr 20**	S-CRC	hsa-mir-1825, hsa-mir-644, hsa-mir-499, hsa-mir-1289-1, hsa-mir-1259, hsa-mir-645, hsa-mir-1302-5, hsa-mir-296, hsa-mir-298, hsa-mir-646, hsa-mir-1257, hsa-mir-1-1, hsa-mir-133a-2, hsa-mir-124-3, hsa-mir-941-1, hsa-mir-941-2, hsa-mir-941-3, hsa-mir-1914, hsa-mir-647
**Chr 22**	UC-P	hsa-mir-649
**Chr X**	UC-P	hsa-mir-651

Note: *present commonly in both UC-P and S-CRC

Table S7: Comparative analysis of all CNVs observed in different sub-groups with the data from The Cancer Genome Atlas Network (TCGA) project for CRC

244k aCGH S-CRC – CNVR data					CGAP data				
Chr	***Start***	***End***	***Size***	***Status***	***Chr***	***Start***	***End***	***Size***	***Status***
chr4	173665872	173666272	1000	***AMP***	chr4	148868312	191273063	42404751	***DEL***
chr8	7358840	7747972	389132	***DEL***	chr8	3609468	39891310	36281842	***DEL***
chr8	39080685	39094953	14268	***DEL***	chr8	3609468	39891310	36281842	***DEL***
chr8	39080685	39094953	14268	***DEL***	chr8	31150731	48817264	17666533	***DEL***
chr8	39356395	39535855	179460	***DEL***	chr8	3609468	39891310	36281842	***DEL***
chr8	39356395	39535855	179460	***DEL***	chr8	31150731	48817264	17666533	***DEL***
chr8	126843399	130486270	3642871	***AMP***	chr8	128222988	129039208	816220	***AMP***
chr12	3455290	5026118	1570828	***AMP***	chr12	2980135	4569646	1589511	***AMP***
chr15	18741516	20220616	1479100	***DEL***	chr15	1	43105206	43105205	***DEL***
chr20	28265913	62379259	34113346	***AMP***	chr20	41763214	42791312	1028098	***AMP***
chr20	28265913	62379259	34113346	***AMP***	chr20	29339506	29741647	402141	***AMP***
chr20	28265913	62379259	34113346	***AMP***	chr20	51575611	62435964	10860353	***AMP***
**244k aCGH UC-P – CNVR data**					**CGAP data**				
chr8	7729111	7747972	18861	***AMP***	chr8	3609468	39891310	36281842	***DEL***
chr8	39356395	39488481	132086	***DEL***	chr8	31150731	48817264	17666533	***DEL***
chr8	39356395	39488481	132086	***DEL***	chr8	3609468	39891310	36281842	***DEL***
chr8	127984937	128919983	935046	***AMP***	chr8	128222988	129039208	816220	***AMP***
chr12	33654	5974091	5940437	***AMP***	chr12	2980135	4569646	1589511	***AMP***
chr15	18657188	20250086	1592898	***AMP***	chr15	1	43105206	43105205	***DEL***
chr15	30422992	30649059	226067	***AMP***	chr15	1	43105206	43105205	***DEL***
chr15	30710135	30710535	400	***AMP***	chr15	1	43105206	43105205	***DEL***
**244k aCGH UC-NP – CNVR data**					**CGAP data**				
chr1	160806847	160813371	6524	***AMP***	chr1	116945608	188847994	71902386	***AMP***
chr4	171984321	172084742	100421	***AMP***	chr4	148868312	191273063	42404751	***DEL***
chr15	18741516	20060261	1318745	***AMP***	chr15	1	43105206	43105205	***DEL***

Note: Chr: chromosome; Size in base pairs; AMP: amplification; DEL: deletion; CGAP: cancer genome atlas project; CNVR-Copy number variation regions

Table S8: Gene set enrichment analysis (GSEA) for S-CRC group CNV associated genes

	Cytokines and growth factors	Transcription factors	Cell differentiation markers	Protein kinase	Translocated cancer genes	Oncogenes	Tumor suppressor
**Tumor suppressor**	0	0	0	0	0	0	1
**Oncogenes**	0	2	0	0	5	**6***	
**Translocated cancer genes**	0	2	0	0	5		
**Protein kinase**	0	0	0	10			
**Cell differentiation markers**	0	0	3				
**Transcription factors**	0	36					
**Cytokines and growth factors**	7						

Note: (6* oncogenes are CCND2, MYC, TOP1, GNAS, MAFB, SS1BL1**)**

Table S9: Gene set enrichment analysis (GSEA) for UC-P group CNV associated genes

	**Cytokines and growth factors**	**Transcription factors**	**Cell differentiation markers**	**Protein kinase**	**Translocated cancer genes**	**Oncogenes**	**Tumor suppressor**
**Tumor suppressor**	0	0	0	0	0	0	0
**Oncogenes**	0	1	0	1	4	**5***	
**Translocated cancer genes**	0	1	0	0	4		
**Protein kinase**	0	0	0	3			
**Cell differentiation markers**	0	0	0				
**Transcription factors**	0	10					
**Cytokines and growth factors**	4						

Note: (5* oncogenes are CCND2, MYC, ERC, MET, KDM5A**)**

Table S10: The functional KEGG pathways enriched with genes located on the chromosomal segments with copy number alterations in S-CRC and UC-P samples

Sample	Pathways	No. of Genes	P Value
UC-P	MAPK signaling pathway	7	0.005652
	Focal adhesion	6	0.007739
	Pathways in cancer	7	0.014941
	Wnt signaling pathway	4	0.068003
	Melanoma	3	0.072888
S-CRC	Viral myocarditis	5	0.036291
	TGF-beta signaling pathway	5	0.057384
	Asthma	3	0.091450
	Pathways in cancer	3	0.092456
	O-Glycan biosynthesis	3	0.097959

Note: UC-P: ulcerative colitis progressors; S-CRC: sporadic colorectal cancer

Table S11: Prediction accuracy of colorectal neoplasia using the 6-gene panel instability signature along with MSI

	Sensitivity (Range)	***Specificity (Range)***	***Overall positive (Frequency)***
S-CRN	54.08 (43-64)	86.66 (69-96)	53/98 (54.1%)
S-CRN with MSI	58.16 (47-68)	86.66 (69-96)	57/98 (58.2%)
UC-CRN	29.03 (14-48)	86.66 (69-96)	9/31(29%)

Table S12: Details of TaqMan CNV assays used in the microarray validation study

Gene	HsID	Chromosomal Location	Dye-Quencher	Product Size	Assays Available
**C-MYC**	Hs02045885_cn	Chr.8:128753146	FAM-NFQ/MGB	100 bp	Pre-designed
**MYCN**	Hs00824796_cn	Chr.2:16080783	FAM-NFQ/MGB	106 bp	Pre-designed
**CCND1**	Hs01425024_cn	Chr.11:69456213	FAM-NFQ/MGB	95 bp	Pre-designed
**CCND2**	MAHE_CNV	Chr.12: 4385847	FAM-NFQ/MGB	102 bp	Custom
**EGFR**	MAHE90017867	chr7:55259406	FAM-NFQ/MGB	89 bp	Custom
**FNDC3A**	Hs06372619_cn	Chr.13:49570034	FAM-NFQ/MGB	109 bp	Pre-designed
**UGT2B17**	Hs04282679_cn	Chr.4:69428283	FAM-NFQ/MGB	99 bp	Pre-designed
**RNaseP**	4403326	Chr14	VIC-TAMARA		Pre-designed

Table S13: Details of antibodies and staining conditions used for Immunohistochemistry (IHC)

Primary antibody	Source	clone	localization	Host	Method
**Ki67**	ScyTek	SP6	Nuclear	Rabbit	ARS + 1 hr incubation
**P53**	ScyTek	DO-7	Nuclear	Mouse	ARS + 1 hr incubation
**EGFR**	Quartett	111.6	Cytoplasm, plasma membrane	Mouse	ARS + 1 hr incubation
**ERBB2**	ScyTek	SP3	Cell membrane	Rabbit	ARS + 1 hr incubation
**Cyclin D1**	Quartett	DCS-6	----	Mouse	ARS + 1 hr incubation
**FNDC3A**	Abcam	M01	Endoplasmic reticulum	Rabbit	ARS + Overnight incubation
**AMACR**	DAKO	13H4	Plasma membrane vesicles	Rabbit	ARS + 1 hr incubation
**C-MYC**	Quartett	9E10.3	Nuclear	Mouse	ARS + 1 hr incubation

Note: ARS: Antigen retrieval system: using microwave and citrate buffer pH 6.0 and 7.4
