# Supplementary material for: Comparative analysis of copy number variations in ulcerative colitis associated and sporadic colorectal neoplasia
Source: BMC Cancer. 2016 Apr 14;16:271. doi: 10.1186/s12885-016-2303-4 (PMC4831153; doi:10.1186/s12885-016-2303-4)
Supplement: Additional file 2: Figure S1. — Overall workflow and design of the study. Figure S2. Common genes found associated with CNVs in UC non-progressor, UC progressor and sporadic colorectal cancers. Figure S3. Enrichment in biological process (GO analysis) of the gene from S-CRC and UC-P samples 244 k aCGH data. X-axis: number of genes involved in the given function and Y-axis: biological function the genes are involved. Figure S4. Results from the screening of gene CNVs (amplification and deletion) in subgroups of sporadic and ulcerative colorectal neoplasm samples in our validation panel of markers by qRT-PCR method. (Abbreviations used are as given earlier). Figure S5. Clustering of qRT-PCR data using 6 genes and 163 samples of different groups. The relative copy number for each gene was plotted against different sample groups from the current study. Figure S6. Summary of Ingenuity Pathways Analysis (IPA) for the role and interaction of the 6 genes markers panel. Figure S7. Results of immunohistochemistry analysis carried out on UC associated and sporadic colorectal cancer samples for various proteins; representative images are for A: p53 B: Cyclin D1; C: AMACR; D: EGFR; E: C-MYC; F: ERBB2; G: Ki67; H: FNDC3A. Figure S8. Box plot illustrating percentage of Ki-67 positive cells in different sample groups of the current study: ulcerative colitis-non progressor (UC-NP) group, ulcerative colitis- progressor (UC-P) group and sporadic colorectal neoplasia (S-CRN) group. (PDF 551 kb) [file 12885_2016_2303_MOESM2_ESM.pdf]

Additional File 1: Figure S1 to S8

Figure S1

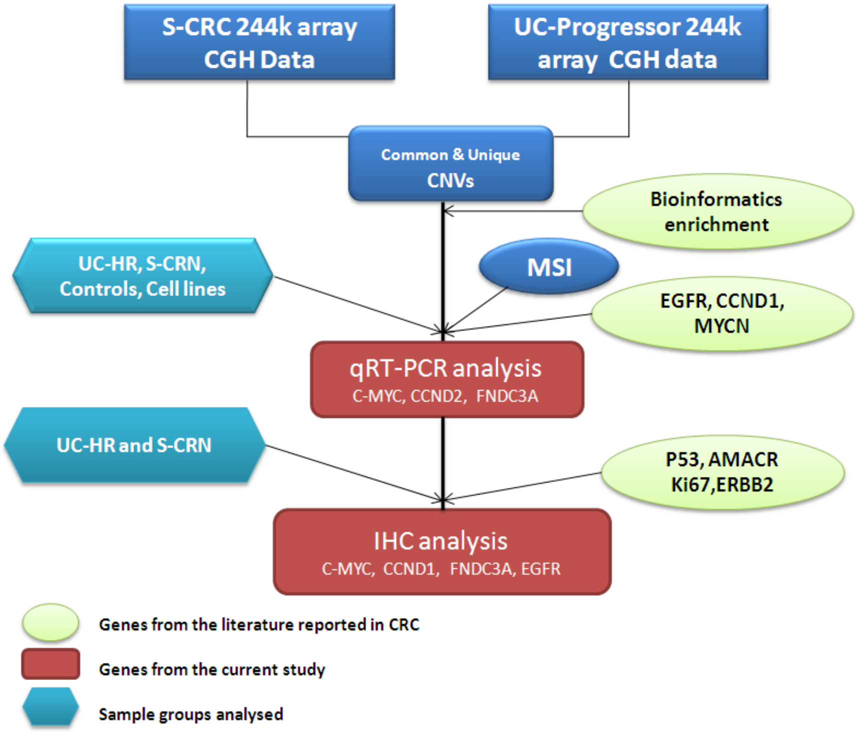

Figure S1: Overall workflow and design of the study.

Figure S2

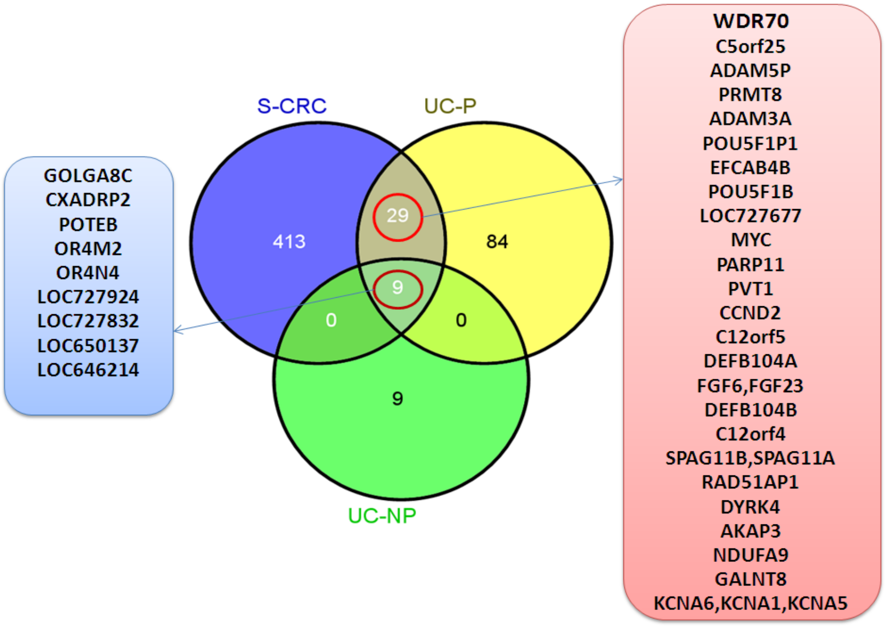

Figure S2: Common genes found associated with CNVs in three groups.

Figure S3

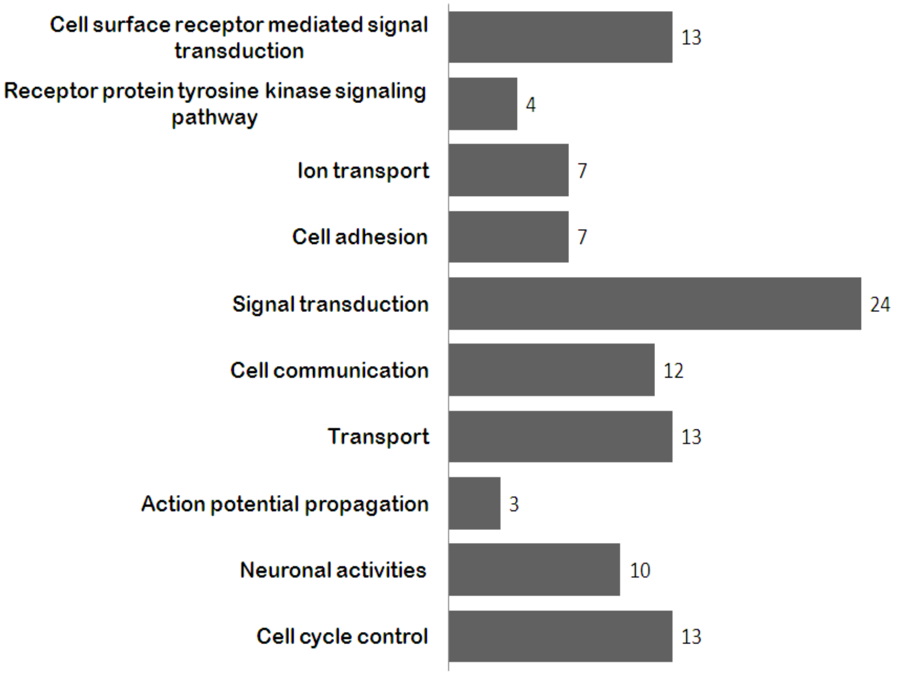

Figure S3: Enrichment in biological process (GO analysis) of the gene lists from S-CRC and UC-P samples 244k aCGH data. X-axis: number of genes involved in the given function and Y-axis: Biological function the genes are involved.

Figure S4

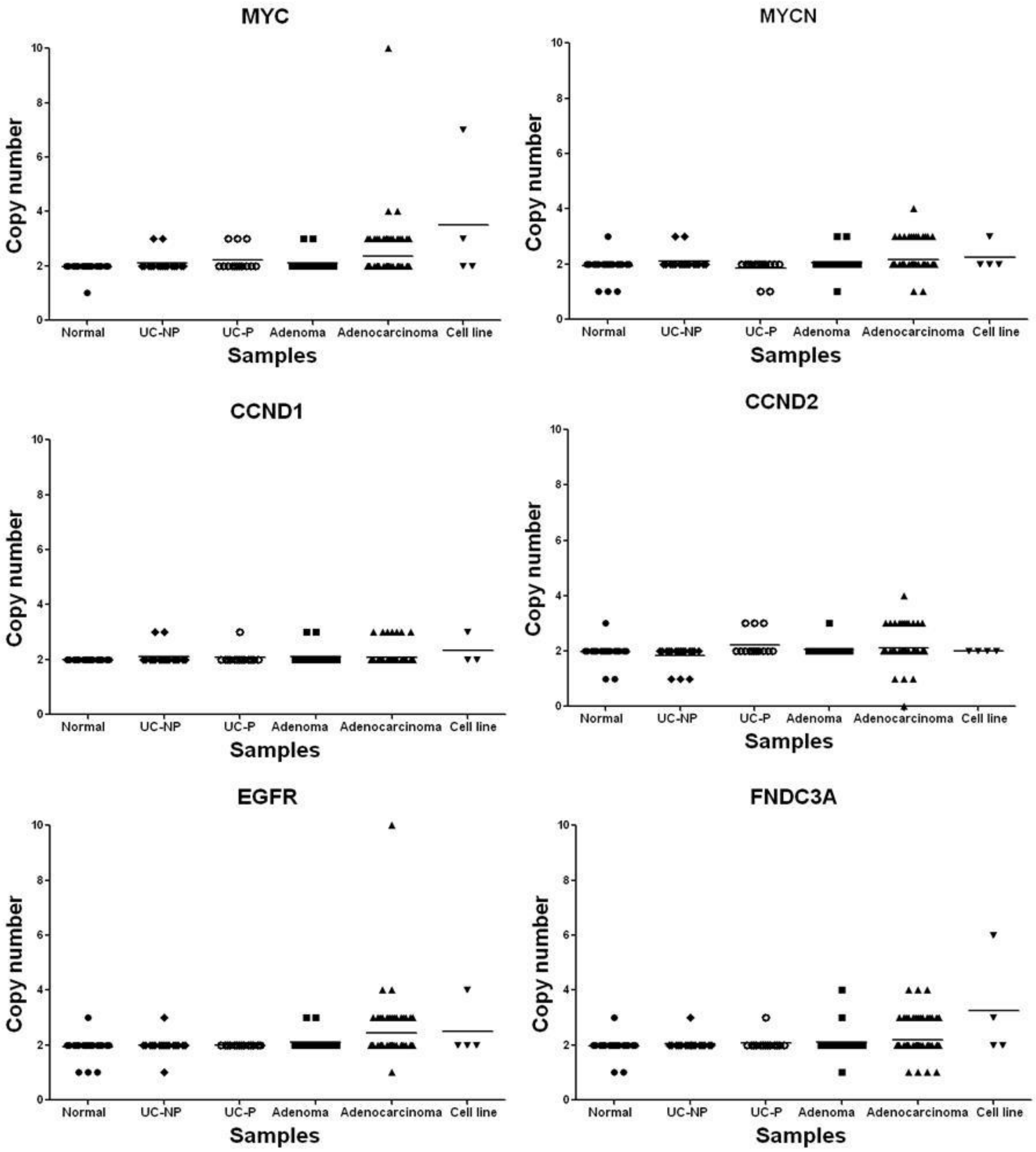

Figure S4: Results from the screening of gene alterations (amplification and deletion) in subgroups of sporadic and ulcerative colorectal neoplasm samples in our validation panel of markers by qRT-PCR method. (Abbreviations used are as given earlier)

Figure S5

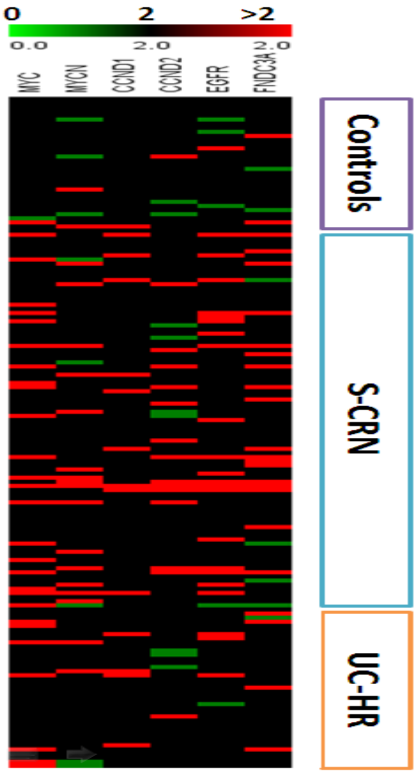

Figure S5: Clustering of qRT-PCR data using 6 genes and 163 samples of different groups. The relative copy number for each gene was plotted against different sample groups in the current study.

Figure S6

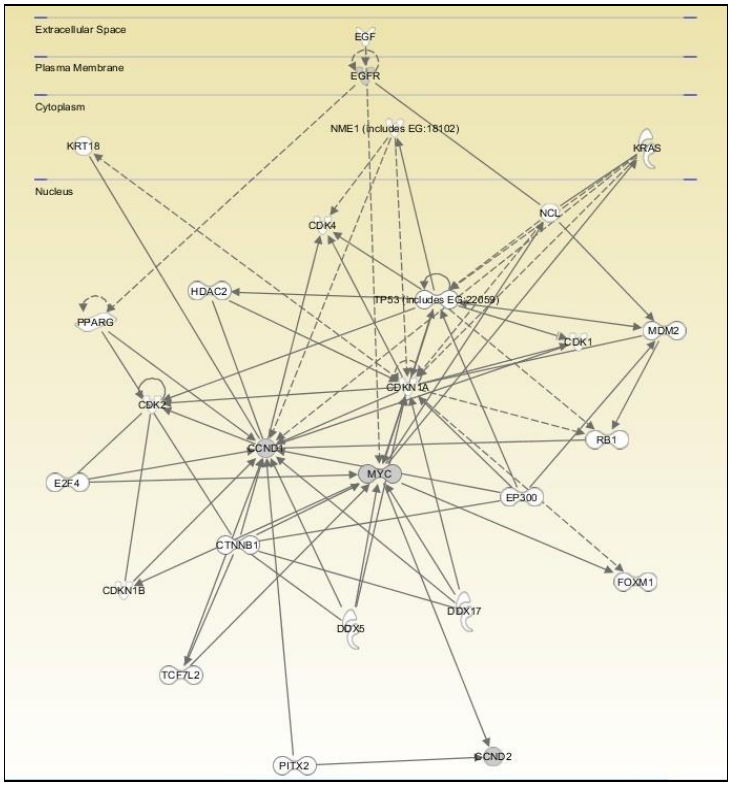

Figure S6: Summary of Ingenuity Pathways Analysis (IPA) for the role and interaction of the 6 genes of the markers panel

Figure S7

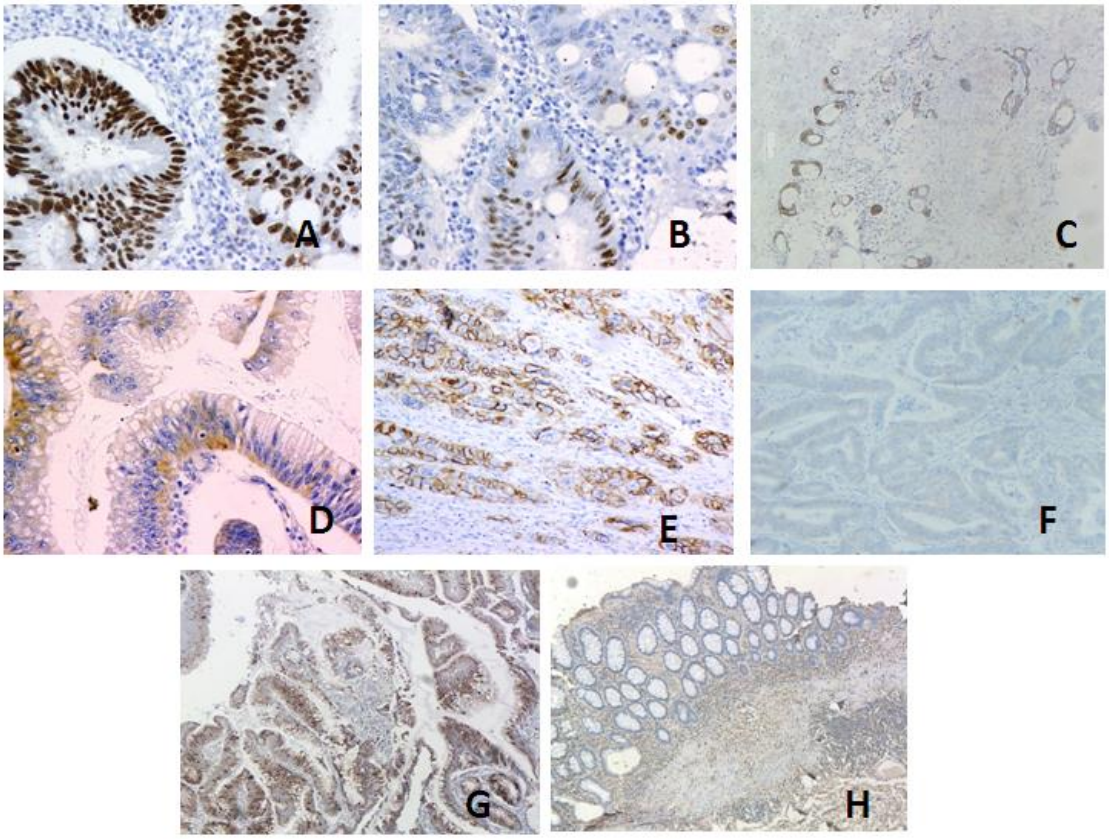

Figure S7: Results of immunohistochemistry analysis carried out on UC associated and sporadic colorectal cancer samples for various proteins; images in order **A:** p53 **B:** Cyclin D1; **C:** AMACR; **D:** EGFR; **E:** C-MYC; **F:** ERBB2; **G:** Ki67; **H:** FNDC3A.

Figure S8

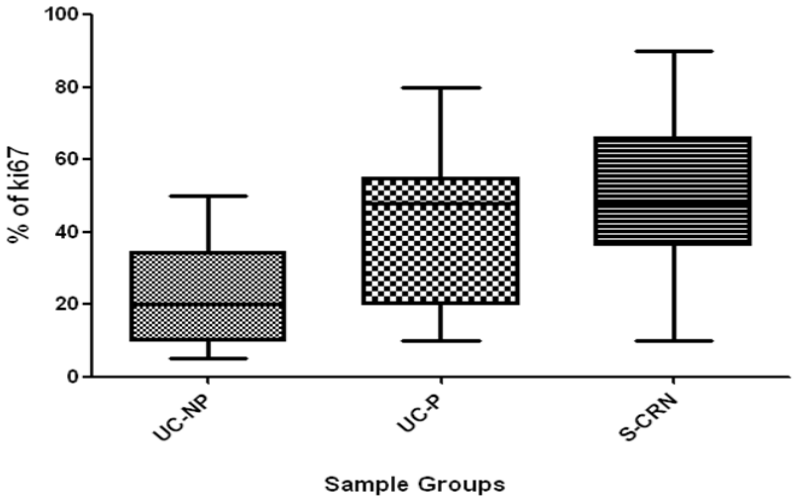

Figure S8: Box plot illustrating percentage of Ki-67 positive cells in different sample groups of the current study: ulcerative colitis-non progressor (UC-NP) group, ulcerative colitis- progressor (UC-P) group and sporadic colorectal neoplasia (S-CRN) group.
